# Fibroblast-derived Gremlin1 localises to epithelial cells at the base of the intestinal crypt

**DOI:** 10.18632/oncotarget.27050

**Published:** 2019-07-23

**Authors:** Louise R. Dutton, Owen P. Hoare, Amy M.B. McCorry, Keara L. Redmond, Noor Eisa Adam, Shannon Canamara, Victoria Bingham, Paul B. Mullan, Mark Lawler, Philip D. Dunne, Derek P. Brazil

**Affiliations:** ^1^Wellcome-Wolfson Institute for Experimental Medicine, Queen’s University Belfast, Belfast, Northern Ireland, UK; ^2^Centre for Cancer Research and Cell Biology, Queen’s University Belfast, Belfast, Northern Ireland, UK; ^3^Mohammed Bin Rashid University of Medicine and Health Sciences, Dubai Healthcare City, United Arab Emirates; ^4^Indonesia International Institute for Life-Sciences, University of East Jakarta, Jakarta Timur, Indonesia; ^*^These authors contributed equally to this work; ^**^Co-senior authors

**Keywords:** Gremlin1, colorectal cancer, fibroblasts, stroma, pathology

## Abstract

Gremlin1 (GREM1) is a secreted glycoprotein member of the differential screening-selected gene in aberrant neuroblastoma (DAN) family of bone morphogenetic protein (BMP) antagonists, which binds to BMPs preventing their receptor engagement. Previous studies have identified that stage II colorectal cancer (CRC) patients with high levels of *GREM1* gene expression in their tumour tissue have a poorer prognosis. Using a series of *in silico* and *in situ* methodologies, we demonstrate that *GREM1* gene expression is significantly higher (*p* < 0.0001) in CRC consensus molecular subtype 4 (CMS4), compared to the other CMS subtypes and correlates (*p* < 0.0001) with levels of cancer-associated fibroblasts (CAFs) within the CRC tumour microenvironment (TME). Our optimised immunohistochemistry protocol identified endogenous GREM1 protein expression in both the muscularis mucosa and adjacent colonic crypt bases in mouse intestine, in contrast to RNA expression which was shown to localise specifically to the muscularis mucosa, as determined by *in situ* hybridisation. Importantly, we demonstrate that cells with high levels of GREM1 expression display low levels of phospho-Smad1/5, consistent with reduced BMP signalling. Taken together, these data highlight a novel paracrine signalling circuit, which involves uptake of mature GREM1 protein by colonic crypt cells following secretion from neighbouring fibroblasts in the TME.

## INTRODUCTION

Gremlin1 (GREM1) is a conserved 184 aa glycoprotein antagonist of bone morphogenetic protein (BMP) signalling which regulates organogenesis and differentiation [1, 2]. GREM1 has also been reported to signal via BMP-independent mechanisms including vascular endothelial growth factor receptor 2 (VEGFR2), Slit proteins and fibrillin [3]. Tight regulation of stem cell homeostasis in the colonic crypt requires polarized, opposing expression gradients of BMP and Wnt (Wingless/Integrated), regulated by BMP antagonists such as GREM1 and Noggin along the crypt vertical axis [4], which is thought to be a key barrier to colorectal tumorigenesis. Consistently, *GREM1* overexpression has been linked to a range of cancers, including colorectal cancer (CRC) [5–10], with mutations affecting BMP signalling occurring in a large number of CRCs [11].

Molecular profiling in CRC has identified four consensus molecular subtypes (CMS) [12]; CMS1 is associated with microsatellite instability (MSI) and a strong immune response, CMS2 and CMS3 are epithelial-rich subtypes, characterised by Wnt signalling and metabolic dysregulation, and CMS4, the “mesenchymal/EMT” subtype, is linked with stromal infiltration, angiogenesis, TGF-β activation and the lowest relapse-free and overall patient survival [12]. Dysregulation of BMP signalling is implicated in disrupted epithelial phenotypes, possibly through epithelial-to-mesenchymal transition (EMT) [13] and cancer cell proliferation [14–16]. Consistently, increased epithelial-specific *GREM1* gene and protein expression is observed in tumour tissue from patients with hereditary mixed polyposis syndrome (HMPS), caused by a single 40-kb genetic duplication on chromosome 15 in the *GREM1* enhancer region [17]. Furthermore, *GREM1* overexpression has also been identified in epithelial cells of more common sporadic colorectal traditional serrated adenomas (TSAs) [18] at the desmoplastic invasive front [13].

In this study, we use *in silico* and *in situ* methods to confirm the prognostic value of *GREM1* and provide a comprehensive assessment of the cellular source of RNA and protein, which may differ from the final protein localisation of GREM1 in the intestine.

## RESULTS

### Clinical relevance of *GREM1* gene expression in CRC patients

**Figure 1:**
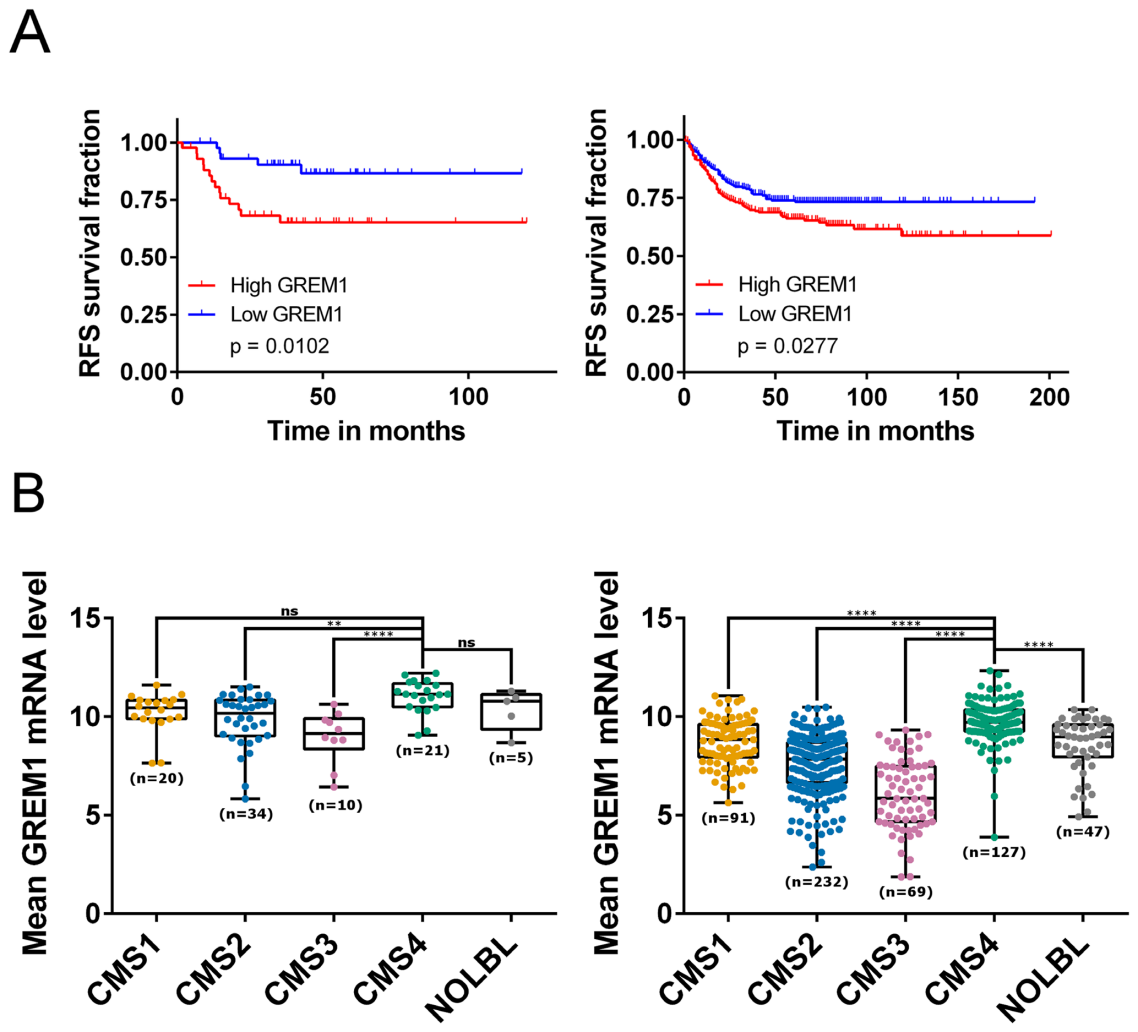
*GREM1* mRNA levels correlate with prognosis, CMS subtypes and cancer cell types. (**A**) Kaplan-Meier curves for GSE33113 (left; *n* = 90, 45 patients per group) which contains data from primary tumour resections of stage II CRC patients, and GSE39582 (right; *n* = 557 (low, *n* = 279; high, *n* = 278), which contains primary tumour resections of stage I-IV CRC patients, showing that patients with high expression (above median; red) of *GREM1* mRNA have a poorer relapse-free survival compared to those with a low expression (blue; *p* = 0.0102 and *p* = 0.0277 respectively). (**B**) Boxplots showing *GREM1* mRNA levels within each of the four CMS subtypes (CMS1-4) together with samples that were unclassified with no CMS label (NOLBL) in GSE33113 (left) and GSE39582 (right). CMS4 patients displayed the highest level of *GREM1* mRNA compared to other subtypes (ns non-significant, ^**^ *p* < 0.01, ^***^ *p* < 0.001, ^****^ *p* < 0.0001).

We confirmed the association between high levels of *GREM1* mRNA and disease recurrence, using a previously described cohort of stage II CRC [19] (GSE33113 [20]; *n* = 90; Figure 1A left), in addition to validation of these findings in a further independent CRC cohort (GSE39582 [21]; *n* = 557; Figure 1A right). Increased levels of epithelial-specific *GREM1* mRNA and protein expression have previously been identified in intestinal tissue from HMPS patients [17]. This predisposition is very rare, therefore we analysed *GREM1* mRNA levels across five non-HMPS CRC transcriptional datasets that had been stratified into CMS subtypes [12], and identified significantly higher quantities of *GREM1* mRNA in the fibroblast-rich CMS4 subtype compared to CMS1-3 (Figure 1B, Supplementary Figure 1).

### Cellular source of *GREM1* gene expression in CRC

**Figure 2:**
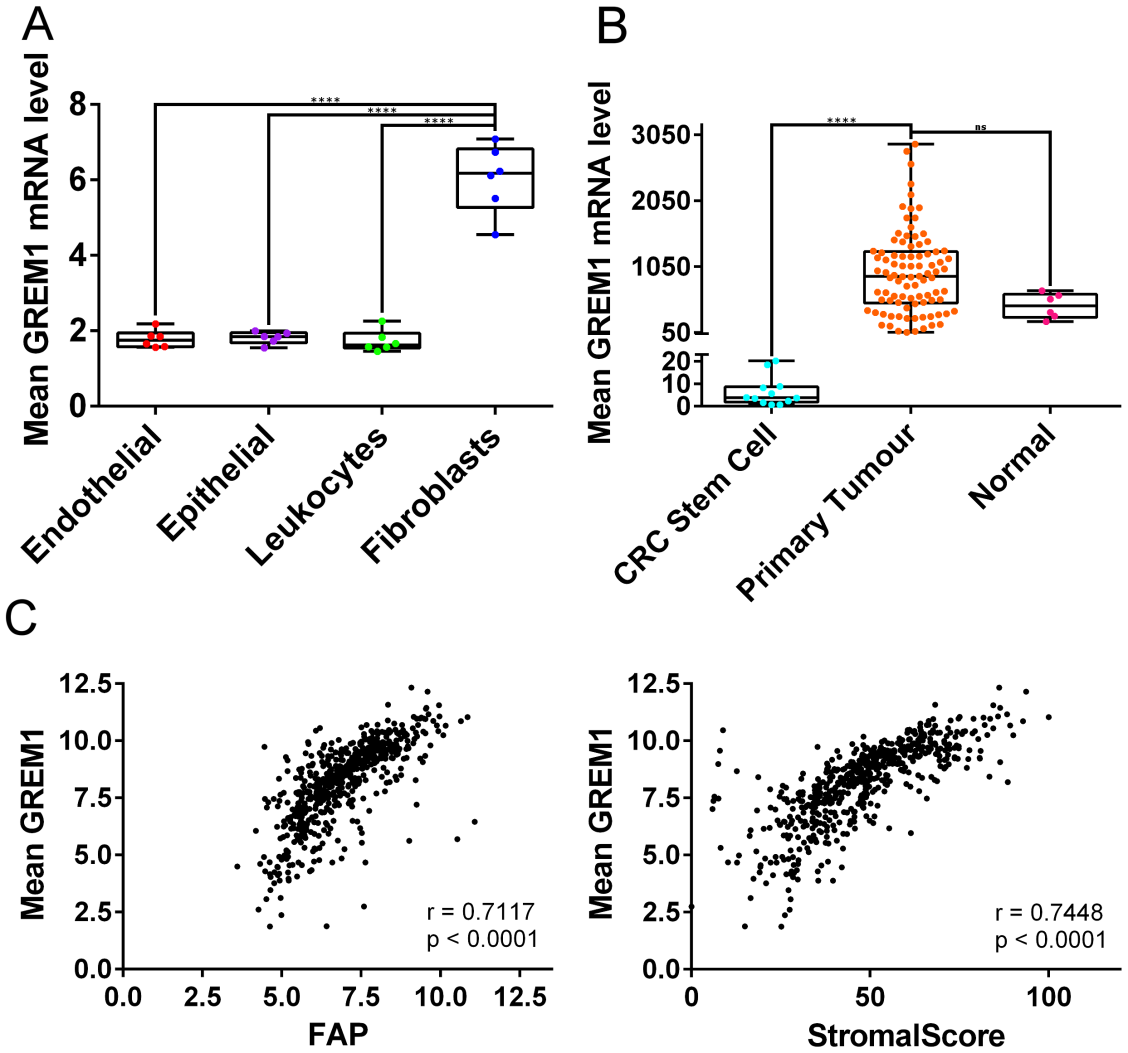
*GREM1* mRNA levels in cell populations and tumour tissue, and correlations with *FAP* expression and StromalScore. (**A**) In GSE39396, which contains data from six fresh colon tumours sorted using fluorescence-activated cell sorting (FACS), fibroblasts contain more *GREM1* mRNA than endothelial cells, epithelial cells and leukocytes (all *p* < 0.0001). (**B**) In GSE33114, *GREM1* mRNA levels are significantly higher in primary tumours compared to CRC stem cells (*p* < 0.0001) and borderline significant compared to normal tissue (*p* = 0.0744). (ns non-significant, ^****^ *p* < 0.0001). (**C**) Scatterplot (left) showing positive correlation between *GREM1* mRNA levels and FAP expression in GSE39582 (Pearson *r* = 0.7117, *p* < 0.0001). *GREM1* mRNA levels also positively correlated with StromalScore (right; Pearson *r* = 0.7448, *p* < 0.0001).

We and others have previously highlighted the association between CMS4, high levels of cancer-associated fibroblasts (CAFs), and poor prognosis in CRC [22–25] Therefore, we next set out to delineate the cellular-specific source of *GREM1* from within the TME of stromal-rich CMS4 tumours. Previously published data suggested that GREM1 protein expression was associated with the epithelial compartment of the TME in HMPS and TSA tissue [19]. In contrast, using a cohort of CRC tissue sorted into purified pools according to their cellular lineages (GSE39396 [26]; endothelial, epithelial, leukocytes and fibroblasts), we now demonstrate that *GREM1* mRNA levels are significantly associated with CAFs compared to other components of the TME (*p* < 0.0001; Figure 2A). In addition, in non-cancer tissue we find that *GREM1* mRNA is higher in smooth muscle cells compared to any other cellular lineage or tissue (Supplementary Figure 2A). Consistent with these data, we demonstrated that *GREM1* mRNA levels are significantly higher in CRC primary tumour tissue, which contains all components of the TME, compared to epithelial stem cells, which are devoid of other TME lineages such as fibroblasts (GSE33114 [20] *p* < 0.0001; Figure 2B). These results were validated in an additional independent cohort (GSE100550 [27]), showing *GREM1* mRNA is significantly higher in CRC tumour tissue compared to either CRC spheroids, organoids or cell lines (all *p* < 0.0001; Supplementary Figure 2B). Furthermore, a direct correlation was demonstrated between *GREM1* mRNA levels and both fibroblast activation protein (*FAP)* mRNA (Pearson *r* = 0.7117, *p* < 0.0001 Figure 2C left) or ESTIMATE StromalScore (Pearson *r* = 0.7448, *p* < 0.0001; Figure 2C right) in transcriptional data from primary CRC tumour tissue (GSE39582 [23]).

### Contrast between Grem1 protein localisation and source of transcript

Other reports have identified *GREM1* expression in basal cell cancer CAFs [28]. Our data strongly supports an association of *GREM1* mRNA expression with the CMS4 subtype of CRC and that fibroblasts are the most likely cell source of *GREM1* in these tumours (Figures 1 and 2). Importantly, these data suggest that the prognostic value associated with *GREM1* gene expression is likely a direct result of its correlation to fibroblast levels within this otherwise poor-prognostic subtype.

**Figure 3:**
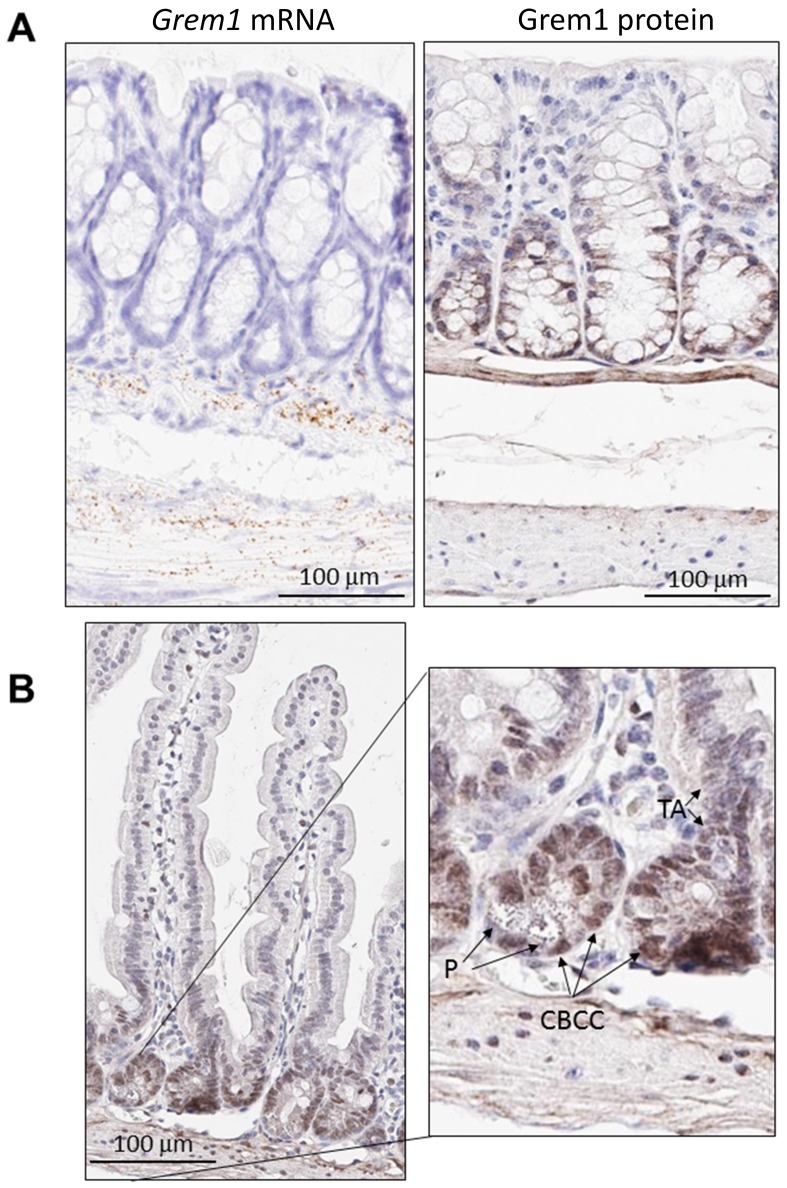
Distinct pattern of endogenous Grem1 expression in mouse colon. Sections (5 μm) from FFPE colon samples (*n* = 4) from wild-type or *Grem1*^-/-^ mice were processed for *in situ* hybridisation (**A** left) and immunohistochemistry (**A** right; **B**.) as described in Methods. Positive *Grem1* mRNA and protein staining was imaged using DAB (brown) and sections were counterstained using haematoxylin and imaged using PathXL. (**A**) *Grem1* mRNA is visible as brown, punctate staining in the muscularis mucosa layer. Scale bars 100 μm. (**B**) Grem1 protein staining is evident as brown staining in the muscularis layer and the base of the colonic crypts (left; scale bar 100 μm). Grem1 protein staining in the musclaris layer as well as cells of the colonic crypt. CBCC, crypt base columnar cells; P, Paneth cells; TA, transit-amplifying cells.

**Figure 4:**
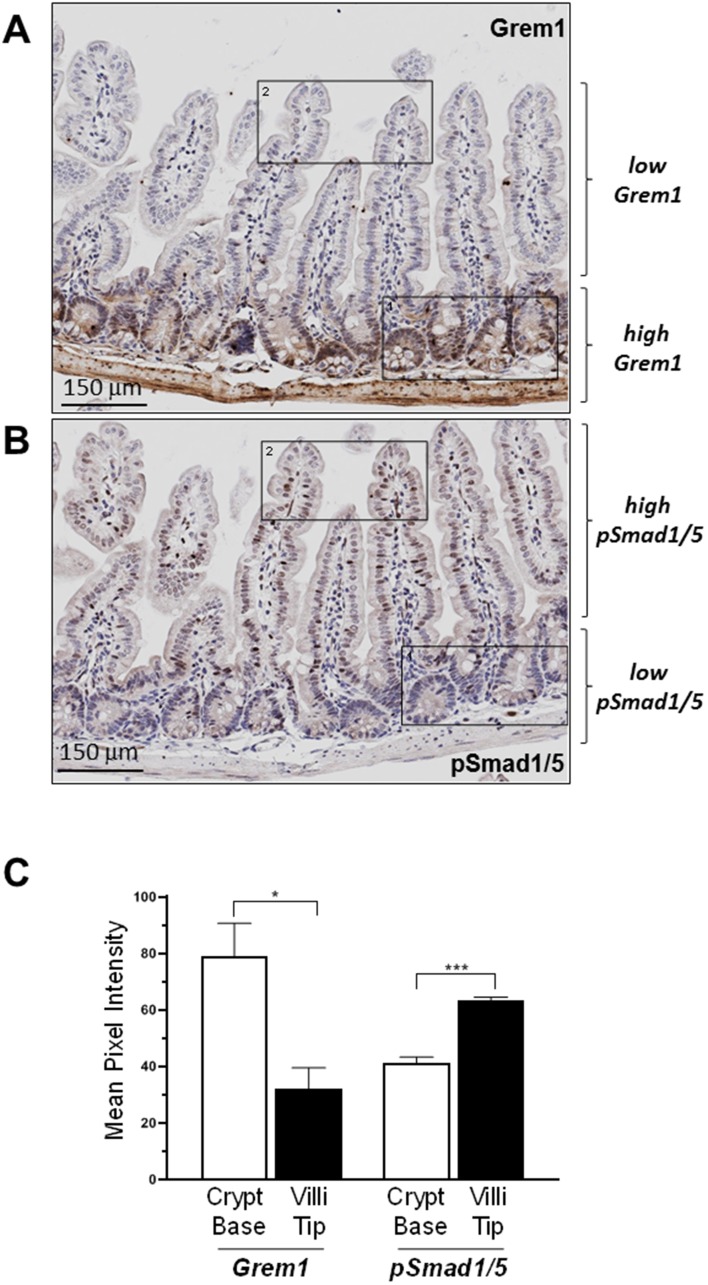
Inverse relationship between expression of Grem1 protein and pSmad1/5 staining in mouse intestine. Sequential FFPE sections (5 μm) of mouse intestine were stained for Grem1 (**A**) or pSmad1/5 (**B**) as described in Methods. (**C**) Positively stained cells (empty bars) or villi tips (filled bars) were quantified in the indicated regions using Image J and data were plotted as mean pixel intensity –/+ SEM (*n* = 3 mice, 3 independent regions of intestine quantified per mouse). ^*^ *p* < 0.05, ^***^ *p* < 0.001 using two-way ANOVA and Bonferroni post hoc test.

Given that GREM1 is a secreted protein that acts as an antagonist of BMP signalling in the extracellular space [3], an immunohistochemistry (IHC) assay was developed to identify the pattern of Grem1 protein expression in mouse colon to be run in parallel with gene expression using RNA *in situ* hybridisation (RNA-ISH). Consistent with our previous data [29] and in line with data presented here (Figure 2), *Grem1* mRNA was almost exclusively localised in the muscularis mucosa in wild-type mice (Figure 3A left). In contrast, Grem1 protein staining was more diffuse, and was detected in both the muscularis mucosa and the base of the colonic crypts (Figure 3A right). Importantly, to fully confirm the specificity of this staining, we utilised intestinal tissue from a *Grem1* genetic knockout mouse model (*Grem1*^-/-^ [29]) (Supplementary Figure 3A and 3B), or IgG isotype controls (data not shown), where no signal was detected for either *Grem1* RNA-ISH or IHC compared to wild-type mice. Further detailed analysis indicated that Grem1 protein was localised in the crypt base columnar cells (CBCC), Paneth cells (P) and transit-amplifying cells (TA) in mouse colon (Figure 3B). Consistent with high levels of Grem1 at the base of the colonic crypts, low levels of pSmad1/5 staining were evident in these cells, indicative of antagonised BMP signalling in this compartment (Figure 4A). In contrast, at the tips of the villi toward the intestinal lumen where Grem1 protein staining was barely detected, levels of pSmad1/5 were higher, suggestive of stronger BMP signalling in these cells (Figure 4B). A significantly higher number of Grem1-positive cells were detected in the crypts versus villi tips, with a reciprocally higher number of pSmad1/5-positive cells detected in the villi tips versus crypts (Figure 4C, Supplementary Figure 4).

## DISCUSSION

**Figure 5:**
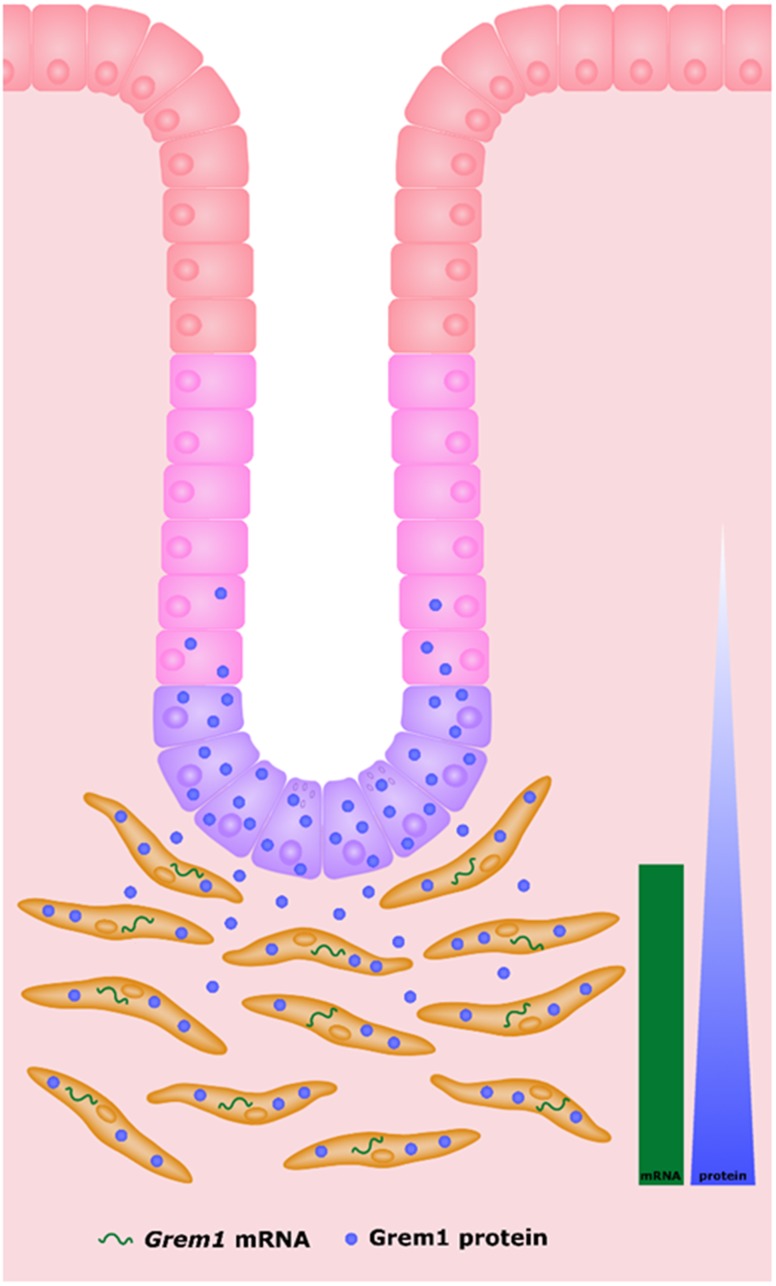
Schematic highlighting the source of *Grem1* mRNA and protein in mouse intestine. *Grem1* mRNA (green) is produced by fibroblasts (orange) in the muscularis layer. Grem1 protein (blue) produced in these cells is then secreted and taken up by cells of the colon crypts (transit-amplifying cells (pink), crypt base columnar cells (purple) and Paneth cells (purple). The levels of Grem1 protein decrease as cells mature up towards the distal epithelial layer (red) nearest the lumen (white).

Comparison of *Grem1* mRNA and protein highlights the differential pattern of staining, where *Grem1* is being transcribed by fibroblasts within the muscularis muscosa, and then secreted from this layer, before finally localising to the cells at the lower levels of the colonic crypt (summarised in Figure 5). Our data indicates a gradient effect, where the highest levels of Grem1 protein are observed at the bottom of the crypt directly proximal to the muscularis muscosa, and becomes more diffuse outside of the crypt base towards the luminal surface. The exact mechanisms of Grem1 protein uptake into colon crypt cells remains to be determined.

In summary, using a number of independent datasets we demonstrate that *Grem1* mRNA predominantly originates in fibroblast/smooth muscle lineages. Following translation to protein, it appears to be secreted and potentially taken up into cells that do not stain for *Grem1* mRNA. Grem1 appears to mediate antagonism of BMP signalling in these cells, based on the low levels of pSmad1/5 detected in the colonic crypts (Figure 4). Data presented here provides a new insight into the mechanism underlying Grem1 signalling in the intestine and provides evidence for the presence of a novel paracrine signalling loop between fibroblasts and epithelial cells to maintain the stem cell niche of the colonic crypt.

## MATERIALS AND METHODS

### Transcriptional analyses

For GSE33113 [20], consisting of stage II primary tumour resections (*n* = 90), and GSE39582 [23], consisting of stage I-IV primary tumour resections (*n* = 557), RMA normalised transcriptional data and relevant clinical information were obtained from Synapse ID syn2623706 (https://www.synapse.org/). For GSE39396 [26], which contains data from four cell populations isolated from fresh colon tumours (*n* = 6), and GSE33114 [20], which contains the primary tumour data from GSE33113 combined with data from colon cancer stem cells (*n* = 12), data was obtained as series matrices from GEO (https://www.ncbi.nlm.nih.gov/geo/). StromalScores were calculated using the estimate package (v1.0.13) [30] in R (v3.5.1).

### Survival analyses, correlations and boxplots

Kaplan-Meier curves with log-rank test, scatterplots with Pearson’s correlation coefficient and boxplots with ANOVA were generated using GraphPad Prism (v6.01; La Jolla, CA, USA).

### *In situ* hybridisation (ISH)

ISH was performed on Formalin-Fixed Paraffin-Embedded (FFPE) sections (5 μm) of wild-type and *Grem1* homozygous genetic knockout (*Grem1*^-/-^) mouse colon, using the RNAscope 2.0 kit (Advanced Cell Diagnostics, Hayward, CA, USA) to detect *Grem1* mRNA (Mm-Grem1, catalog no. 314741) [29]. Slides were scanned using an Aperio AT2 scanner and uploaded to the PathXL digital pathology suite (Belfast, Northern Ireland, UK).

### Immunohistochemistry (IHC)

IHC was performed on wild-type and *Grem1*^-/-^ colon using the VectaStain Goat IgG kit according to manufacturer’s instructions (Vector Laboratories, Burlingame, CA, USA). Sections (5 μm) were deparaffinised on a Leica Bond RX system. Sections were washed and then epitope retrieval performed. Sections were washed in PBS-0.1% Triton-X, quenched with 3% (v/v) H_2_O_2_ in PBS-0.1% Triton-X for 10 min at RT, washed, blocked with 10% (v/v) rabbit serum in PBS-0.1% Triton-X for 60 min at RT in a humidified, dark chamber, and incubated with anti-Grem1 antibody (R&D Systems AF956, raised in goat, 1:250 in PBS-0.1% Triton-X,) or anti-pSmad1/5 Ser463/Ser465 antibody (Thermo Fisher Scientific 31H14L11, raised in rabbit, 1:175 in PBS-0,1% Triton-X) overnight at 4°C. Goat IgG and rabbit IgG isotype controls were also included at the same dilutions. Following washing with PBS-0.1% Triton-X, secondary anti-goat biotin antibody (1:250) was added for 90 min at RT. After washing, the Avidin Biotin Complex (ABC) reagent was added for 30 min at RT. Detection was performed using 3,3’-diaminobenzidine kit (DAB Substrate Kit, SK-4100, Vector Laboratories) for 3–7 min, and reactions were stopped using ddH_2_0. Slides were counterstained on a Sakuara Autostainer using a standard protocol and haematoxylin for 30 sec, and a coverslip added. Slides were scanned using an Aperio AT2 scanner and uploaded to PathXL digital pathology suite.

### Quantification of immunohistochemistry staining

Sequential FFPE sections (5 μm) were stained for Grem1 or pSmad1/5 as above. Images were captured on PathXL and quantification of Grem1 and Smad1/5 staining was carried out on programmes Fiji [31] and ImageJ [32]. Cells were selected within the defined area and the Multi-Point tool was used to calculate Mean Pixel Intensity for each cell. Three mice per group were analysed, with three independent images quantified per mouse. Data was analysed on GraphPad Prism (V8.1.0) and statistical significance determined using two-way ANOVA with Bonferroni post hoc test.

## SUPPLEMENTARY MATERIALS


