# Supplementary material for: Fibroblast-derived Gremlin1 localises to epithelial cells at the base of the intestinal crypt
Source: Oncotarget. 2019 Jul 23;10(45):4630–9. doi: 10.18632/oncotarget.27050 (PMC6659803; doi:10.18632/oncotarget.27050)
Supplement: Supplementary file 1 [file oncotarget-10-4630-s001.pdf]

Fibroblast-derived Gremlin1 localises to epithelial cells at the base of the intestinal crypt

SUPPLEMENTARY MATERIALS

Transcriptional analyses

For GSE14333 [1], containing data from Duke's stage A-D tumour resections (*n* = 157), GSE17536 [2], containing data from stage I-IV colon tumours (*n* = 177) and TCGA data for stage I-IV tumours (*n* = 263), RMA normalised transcriptional data and relevant clinical information were obtained from Synapse ID syn2623706 (https://www.synapse.org/). GSE100550, which contains data from colon cancer cell lines, primary cell lines and human tumour was obtained as a series matrix from GEO (https://www.ncbi.nlm.nih.gov/geo/).

Cell-specific gene expression

GREM1 cell-specific expression was assessed using the BioGPS application [3] (http://biogps.org/gene/26585/) and the GeneAtlas U133A gcrma data [4] which contains from a range of cell types from 79 human and 61 mouse tissues.

REFERENCES

1. Jorissen RN, Gibbs P, Christie M, Prakash S, Lipton L, Desai J, Kerr D, Aaltonen LA, Arango D, Kruhøffer M, Orntoft TF, Andersen CL, Gruidl M, et al. Metastasis-Associated Gene Expression Changes Predict Poor Outcomes in Patients with Dukes Stage B and C Colorectal Cancer. Clin Cancer Res. 2009; 15:7642–7651. https://doi.org/10.1158/1078-0432.CCR-09-1431. [PubMed]
2. Smith JJ, Deane NG, Wu F, Merchant NB, Zhang B, Jiang A, Lu P, Johnson JC, Schmidt C, Bailey CE, Eschrich S, Kis C, Levy S, et al. Experimentally derived metastasis gene expression profile predicts recurrence and death in patients with colon cancer. Gastroenterology. 2010; 138:958–68. https://doi.org/10.1053/j.gastro.2009.11.005. [PubMed]
3. Wu C, Jin X, Tsueng G, Afrasiabi C, Su AI. BioGPS: building your own mash-up of gene annotations and expression profiles. Nucl Acids Res. 2016; 44:D313–D316. https://doi.org/10.1093/nar/gkv1104. [PubMed]
4. Su AI, Wiltshire T, Batalov S, Lapp H, Ching KA, Block D, Zhang J, Soden R, Hayakawa M, Kreiman G, Cooke MP, Walker JR, Hogenesch JB. A gene atlas of the mouse and human protein-encoding transcriptomes. Proc Natl Acad Sci U S A. 2004; 101:6062–7. https://doi.org/10.1073/pnas.0400782101. [PubMed]
5. Linnekamp JF, Hooff SR van, Prasetyanti PR, Kandimalla R, Buikhuisen JY, Fessler E, Ramesh P, Lee KAST, Bochove GGW, de Jong JH, Cameron K, Leersum RV, Rodermond HM, et al. Consensus molecular subtypes of colorectal cancer are recapitulated in *in vitro* and *in vivo* models. Cell Death Differ. 2018; 25:616–633. https://doi.org/10.1038/s41418-017-0011-5. [PubMed]

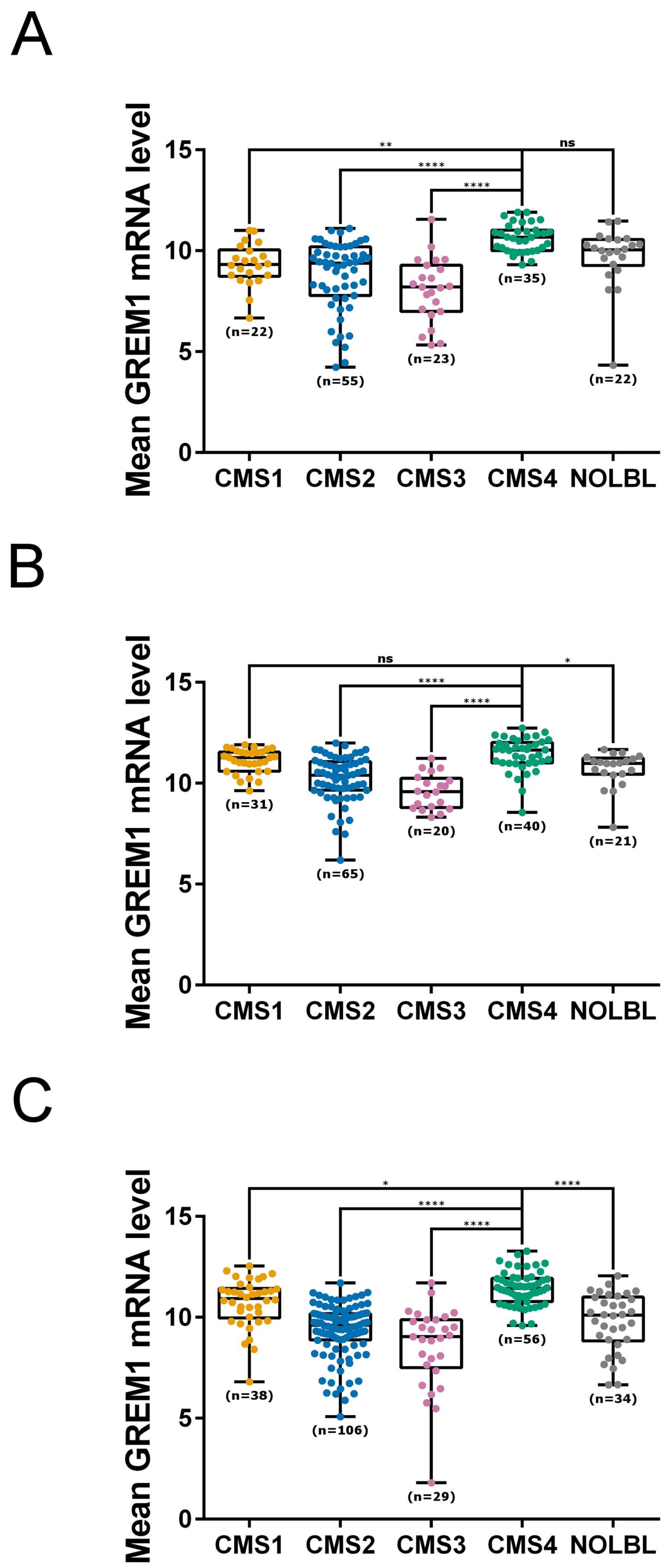

Supplementary Figure 1: *GREM1* expression correlates with the CMS4 subtype of CRC. *GREM1* mRNA levels assessed in (**A**) GSE14333, (**B**) GSE17536 and (**C**) TCGA datasets were compared between the four CMS subtypes (CMS1-4) alongside samples which remained unclassified with no CMS label (NOLBL). CMS4 patients displayed the highest level of *GREM1* mRNA compared to other subtypes (ns non-significant, $^{*}p < 0.05$, $^{**}p < 0.01$, $^{****}p < 0.0001$).

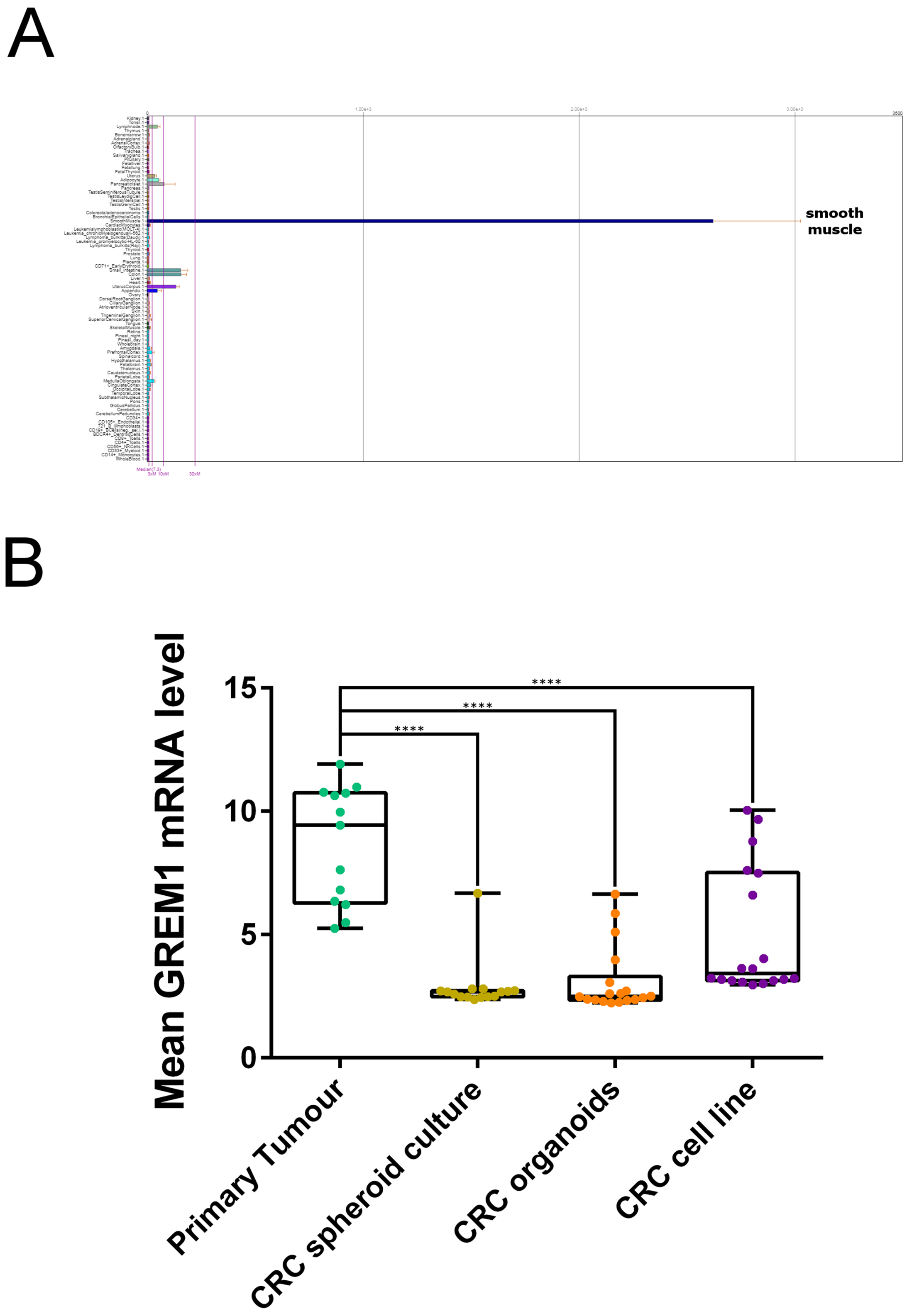

Supplementary Figure 2: *GREM1* expression is significantly higher in smooth muscle cells and CRC tumour tissue. (**A**) *GREM1* mRNA expression is significantly higher in smooth muscle cells compared to any other cellular lineage of tissue in the GeneAtlas U133A, gcrma dataset, which includes a panel of 79 human and 61 mouse tissues [4]. (**B**) *GREM1* mRNA expression is significantly higher in CRC tumour tissue compared to either CRC spheroids, organoids or cell lines (GSE100550 [5] all $p < 0.0001$).

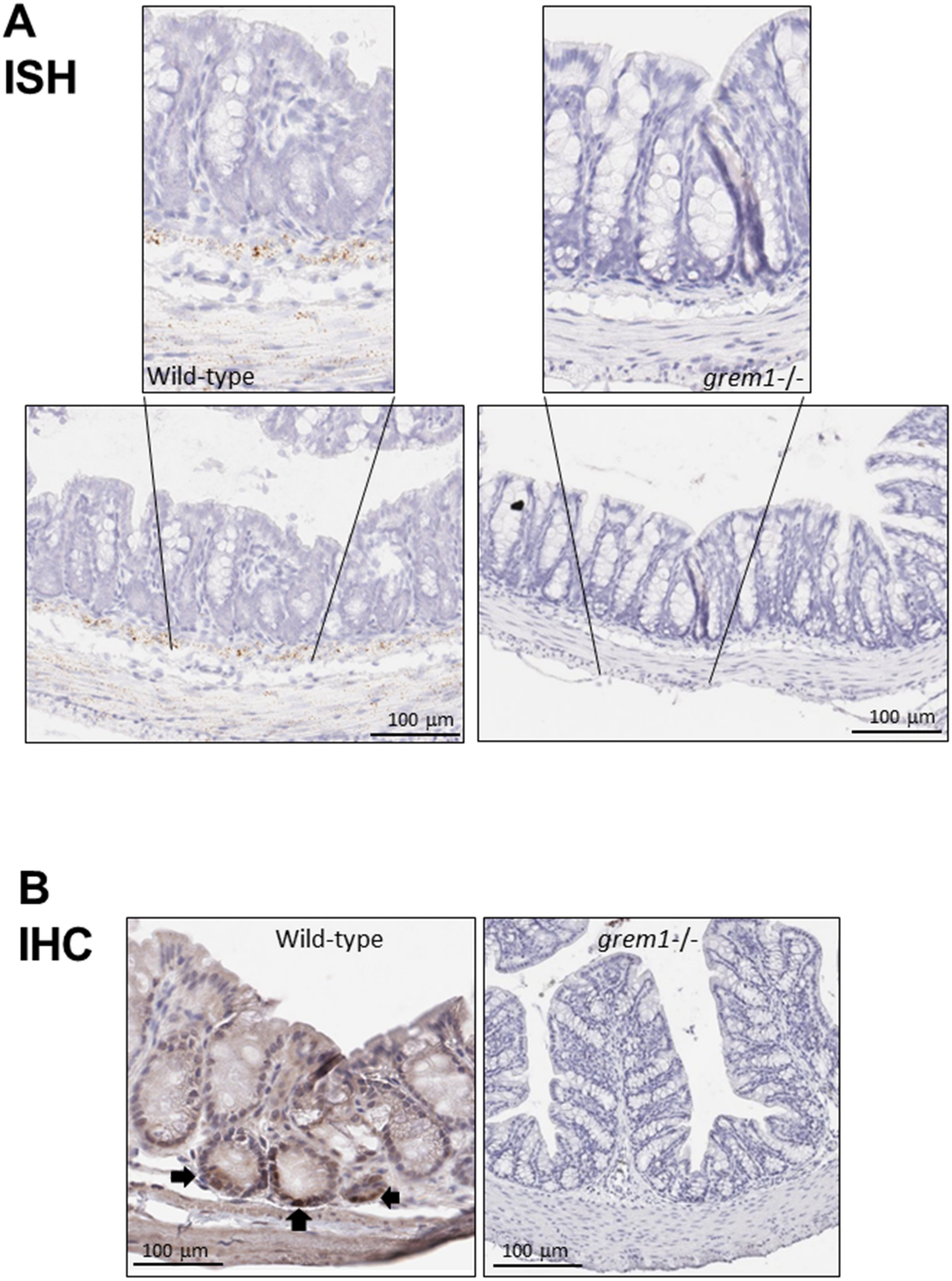

Supplementary Figure 3: *Grem1* mRNA and protein localisation in mouse colon. Sections (5 µm) from FFPE colon samples (*n* = 4) from wild-type or *Grem*$1^{-/-}$ mice were processed for *in situ* hybridisation (**A**) and immunohistochemistry (**B**) as described in Methods. Positive *grem1* mRNA and protein staining was imaged using DAB (brown) and sections were counterstained using haematoxylin and imaged using PathXL. (**A**) *Grem1* mRNA is visible as brown, punctate staining in the muscularis mucosa layer of wild-type mice (left) but not *grem1*$^{-/-}$ mice (right). Scale bars 100 µm. (**B**) Grem1 protein staining is evident as brown staining in the muscularis layer and the base of the colonic crypts of wild-type mice (upper panel). In contrast, no staining is detected in sections from *Grem1*$^{-/-}$ mice (bottom panel). Cells staining positive for Grem1 are indicated by arrows. Scale bars, 100 µm.

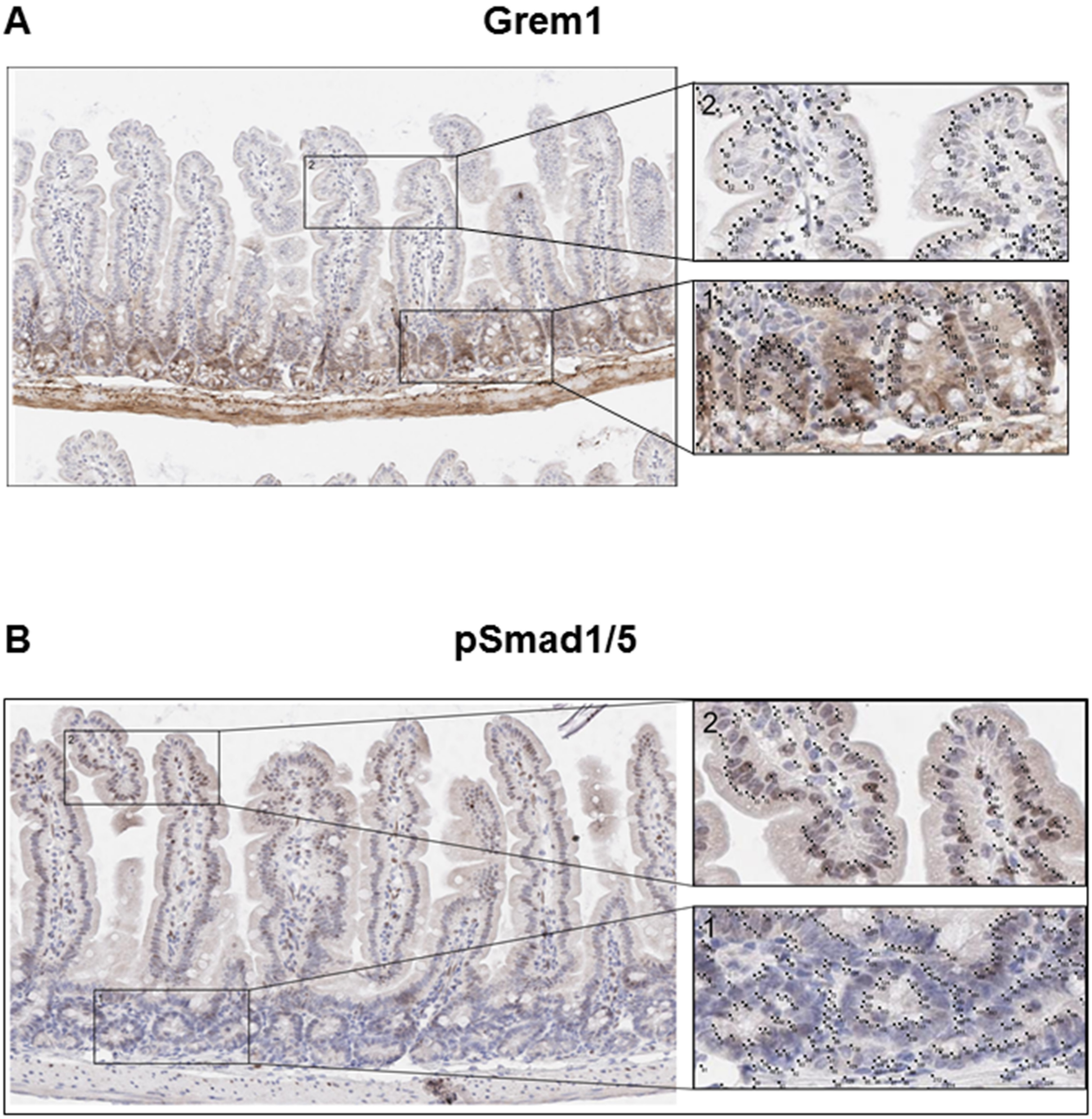

Supplementary Figure 4: Images demonstrating quantification of Grem1-positive or pSmad1/5 positive staining in mouse intestine. Cells were selected in the defined region as described in Methods. Mean pixel intensity per cell was then quantified using Image J and the data plotted. Representative images used for quantitation are shown. (**A**) pSmad1/5 staining in crypt base, (**B**) pSmad1/5 staining at villi tip.
